# Case Report: Occupational Dust Exposure Among Bakery Workers in Perth, Western Australia

**DOI:** 10.3389/fpubh.2021.723154

**Published:** 2021-08-19

**Authors:** Krassi Rumchev, Yun Zhao, Andy Lee

**Affiliations:** School of Population Health, Curtin University, Perth, WA, Australia

**Keywords:** Australia, case report, industrial bakery, respirable dust, flour dust

## Abstract

Occupational dust exposure can occur in various settings, including bakeries. A case study was conducted in an industrial bakery in Perth, Western Australia, to assess exposure to particulate dust concentration. The factory was separated into three production zones and an office area which represented as a control zone. Results indicated that bakery workers in the production zones were exposed to higher ambient dust particle concentrations compared to those from the office environment. Coarse particles (>10 μm in aerodynamic diameter) were the predominant particle size fraction measured in all studied areas with the highest median exposure level recorded in the dough room (0.181 mg/m^3^, interquartile range 0.283). High personal concentration of respirable particles was also measured in the dough room (median 2.26 mg/m^3^) which exceeded the recommended limit of 1.5 mg/m^3^ and was more than 50 times higher than the concentration recorded in the office (0.04 mg/m^3^). The variation in dust concentrations between production zones underlines the need of more knowledge about how aerosol fractions are distributed across the production process. The findings also suggest that bakery workers are exposed to high dust levels that may increase their risk of developing respiratory diseases and the decrease of present exposure levels is imperative.

## Introduction

Exposure to occupational dust can occur in various settings and without appropriate safety precautions the exposure can lead to adverse health outcomes. According to recent research, the most severe exposure to flour dust is usually observed in bakeries and grain mills due to the character of occupational activities in such settings (1).

Occupational dusts are a type of airborne particulate matter defined as “solid particles generated and dispersed into the air” (2). Particle size, measured as aerodynamic diameter, is critical in determining the percentage of dust that enters a worker's respiratory tract and the site at which it deposits (2, 3). Deposition models have concluded that coarse particles ≥10 μm in diameter are more likely to impact onto the surfaces of the upper airways. Fine particles between 5 and 10 μm in diameter are more likely to reach deeper into the respiratory tract and cause adverse respiratory health effects (4–8).

Flour dust has a bimodal distribution reaching its peaks around 5 and 15–30 μm for fine and coarse dust particles, respectively (9). Approximately 50% of the airborne flour dust particle mass has an aerodynamic diameter over 15 μm; however, in dusty areas, particles with smaller aerodynamic sizes ≤ 5 μm can be more prevalent (10).

Research has shown that flour dust exposure is associated with a range of respiratory symptoms, including cough, wheeze, shortness of breath (dyspnea), asthma, eye problems, conjunctivitis, rhinitis, and sinusitis (11, 12). Exposure at 1.35–3.57 mg/m^3^ has been identified with changes in lung function and an increased prevalence of respiratory and asthmatic symptoms (13, 14). It has been reported that respirable flour dust particles smaller than 5 μm potentially caused hypersensitivity pneumonitis and those particles with size between 5 and 10 μm could provoke asthma (1). Asthma among bakery workers may also occur as a result of immunological sensitization following exposure to wheat allergens, and in particular Aspergillus derived α-amylase or trypsin, which are often present in flour dust (15, 16). Exposure to Aspergillus is reportedly a causative agent for invasive infections in immune-compromised individuals, with most cases of azole-resistant disease (17).

A wide range of Occupational Exposure Limits (OEL) exist in different jurisdictions, from 0.5 to 10 mg/m^3^ including the American Conference of Governmental Industrial Hygienist Threshold Limit Value-Time-Weighted Average of 3 mg/m^3^ for exposure to respirable dust (18). The EU Scientific Committee on Occupational Exposure Limits has recommended OEL ≤1.5 mg/m^3^ of respirable dust to protect bakery workers, whereas symptoms in the lower respiratory tract, including asthma and sensitization, are rare in the range 0.5–1.0 mg/m^3^ (19). However, there is enough evidence to show that even exposure levels <1 mg/m^3^ can trigger symptoms in already sensitized workers (20–22). Some studies have reported that there is no safe level of flour dust exposure (23).

The Australian National Hazard Exposure Workers Surveillance, conducted by SafeWork Australia (24), showed that 39% of Australian workers are exposed to airborne hazards, with 80% of them exposed to dust at their place of work. Furthermore, hazardous occupational airborne dust exposure is estimated to affect 31% of workers in Australia (24). A recent survey of 4,878 Australian workers found 8.7% of women and 3% of men were exposed to flour dust at the workplace. Extrapolating to the entire workforce, almost 600,000 workers (358,900 women and 239,400 men) are potentially exposed to flour dust in Australia (25). Despite this, there has been no flour dust monitoring in Australian bakeries. Therefore, the present case study aimed to quantitatively assess the exposure and size distribution of dust particles in an Australian industrial bakery setting.

## Materials and Methods

### Study Setting

This study was conducted in a large industrial bakery located in Perth, Western Australia. The factory is one of the oldest and largest bakeries (> 50 workers) within the Perth metropolitan area and can be considered as a representative of this industry in Western Australia. This food production factory processes raw ingredients to make pastry-based products such as pies and sausage rolls. The factory consists of three working zones, separated by large doors or roller doors. The production of pastry from raw products is performed in the dough room or Work Zone 1 (WZ1). Dough making involves weighing of ingredients, transferring of flour and other baking ingredients to the kneading trough a mixer, and mixing the flour with other ingredients. This process includes loading the flour and raw ingredients into a large stainless-steel tub either by tipping them out of a bag or by use of handheld tools. Once all ingredients are mixed, the tub is transferred to Work Zone 2 (WZ2) to prepare the uncooked pies. The process is highly automated, transferring pastry from containers onto a moving flatbed and then rolled by machines. Work Zone 3 (WZ3) is responsible for the production of pre-cooked sausage rolls and pies and operates on a similar basis to WZ2. People working in this zone are expected to pull away the excess dough and manually add flour or other dust emitting products. During this process, flour is continuously dusted on top of the pastry by special technologies. An adjacent office environment was selected to be the control area.

### Data Collection

All workers (*n* = 29) from the three production zones have signed a consent form for the flour dust monitoring, of whom four were from WZ1, 16 from WZ2, and nine from WZ3. All 10 office workers also agreed to participate in the study. Ethics approval was obtained from the Curtin University Research Ethics Committee.

### Airborne Dust Measurements

Dust measurements were conducted on a randomly selected workday and when convenient for the company. The measurements comprised of (1) monitoring the ambient background concentrations of particulate matter (PM) with different sizes and (2) personal exposure to respirable dust particles.

Real time ambient concentration of PM was conducted in each work zone and office using the TSI DustTrak_TM_ Aerosol Monitor Model 8530 and can measure aerosol concentration between 0.001 and 400 mg/m^3^. The monitor operated at a flow rate of 1.7 L/min and particles with different size fraction including PM_10_, PM_4_, PM_2.5_, and PM_1_ were recorded. In addition to the regular annual factory calibration, the DustTrak™ was custom calibrated using the integral 37 mm filter at selected site locations to determine the gravimetric concentration. The custom calibration factor was reused at all measurement sites. The DustTrak was set up at a height of ~1.5 m above the ground. The equipment was pre-recorded to take measurements every 5 min.

One worker from each area was randomly selected to participate in the personal exposure assessment of respirable dust. Concentration of respirable particles was performed using the SKC Universal sampling pump, following the Australian Standard Method (AS 2985-2009) (26) for sampling and gravimetric determination of respirable dust. The monitor operated at a flow rate of 2.2 L/min. Before each test, the SKC pump was calibrated and charged, and the cyclone was made ready with the filter paper inside. Filter papers were desiccated for 24 h to remove any moisture before and after each sampling. Filters were also weighed before and after sampling using an Advanced Analytical Balance to determine the dust concentration following the Australian Standard (AS 2985-2009) (2). The ambient and the corresponding personal dust exposure monitoring were conducted simultaneously for 8 hours on the same working day between 8 am and 4 pm. All workers were asked to maintain their usual behavioral patterns during sampling.

### Statistical Analysis

All statistical analyses were performed using the IBM SPSS Statistics for Windows, Version 26 (Armonk, NY: IBM Corp). PM with different size concentrations were presented in terms of median and interquartile range (IQR) due to their skewness. The overall difference in PM between the four zones was assessed by the Kruskal-Wallis test. Two-way pairwise group differences using sequential Mann-Whitney tests were applied after detecting significant overall differences (*p* < 0.05).

## Results

Majority of the workers were male (67%), aged between 20 and 45 years (64%) and non-Caucasian (67%). Most of them (74.4%) worked part- time (≤40 h/week) and were non-smokers (56.4%).

### Dust Sampling

**Figure 1:**
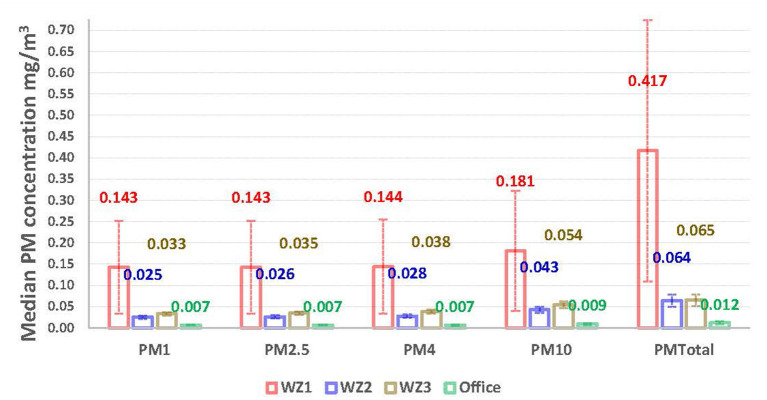
Exposure levels (median with interquartile range) of ambient particulate matter concentrations.

The median ambient PM concentrations measured in the bakery factory varied from 0.004 to 6.220 mg/m^3^ and no values below the detection limit were recorded during sampling. Overall, the PM concentrations measured in the production zones were significantly higher (*p* < 0.001) than those recorded in the office environment. The dustiest section was WZ1 and the least dusty was the office (Figure 1). On average, the median total ambient PM concentration recorded in WZ1 (0.417 mg/m3, IQR 0.615) were significantly higher (*p* < 0.001) than the exposure levels in WZ2 (0.064 mg/m^3^, IQR 0.028), WZ3 (0.065 mg/m^3^, IQR 0.026) and the office (0.012 mg/m^3^, IQR 0.008) (Figure 1).

As shown in Figure 1, coarse particles (≤10 μm in aerodynamic diameter) were the predominant size fraction measured in all studied areas. Above the WHO standard of 0.050 mg/m^3^ (27) were recorded for ambient concentration of PM_10_ in WZ1 (0.181 mg/m^3^, IQR 0.283) and WZ3 (0.054 mg/m^3^, IQR 0.015). Median concentrations of fine particles (PM_2.5_) in the production zones also exceeded the WHO air standard (24) of 0.025 mg/m^3^ with the highest concentration recorded in WZ1 (0.143 mg/m^3^, IQR 0.219). The study found a significant (*p* < 0.001) and strong association (*r* = 0.993) between PM_2.5_ and PM_10_. In addition to the median ambient PM exposures, the study also measured short term peak concentrations (lasting <4 min) in each work zone. For total PM, the highest recorded peak level was observed in WZ1 (6.22 mg/m^3^), followed by WZ3 (1.06 mg/m^3^) and WZ2 (0.238 mg/m^3^).

The results from the ambient dust measurements were consistent with those recorded from the personal sampling of respirable particles, with the highest respirable dust concentration reported in WZ1 (2.26 mg/m^3^), which exceeded the EU's recommended OEL of 1.5 mg/m^3^. Bakery workers who were involved in activities in WZ2 and WZ3 were exposed to lower personal exposure levels of respirable dust, 0.17 and 0.21 mg/m^3^, respectively. The exposure level of respirable particles recorded in the office was 0.04 mg/m^3^.

## Discussion

The results of environmental monitoring in all work zones showed the ambient concentration of total particle dust within the range between 0.004 and 6.220 mg/m^3^. Factory workers in the production zones were exposed to higher ambient concentration of total particle dust when compared with those in the office environment. The highest median ambient concentration for total PM was recorded in WZ1 (0.417 mg/m^3^). This result was consistent with the findings from personal exposure assessments to respirable particles with the highest concentration also measured in WZ1 (2.26 mg/m^3^) which can be explained with the fact that workers in this production zone were exposed to dustiest tasks. Such exposure levels appeared to be comparable or lower than those found in other countries. For example, the total ambient dust concentration of 0.33 mg/m^3^ was reported among 68 bakers in Norway (28), whereas a concentration of 1.56 mg/m^3^ was recorded from flourmills in Iran (29). Moreover, significantly higher levels of dust were evident for dough makers in comparison to those working in other areas of a mill factory (12) which is consistent with the findings of the present case study and was also reported elsewhere (11, 30, 31).

Such levels of exposure to particulate air pollution might lead to a higher prevalence of respiratory symptoms among bakery workers. Ijadunola et al. (12) noted that cough (40%) and sputum production (59%) were more prevalent in the production zones of a bakery compared with the control areas, 4 and 3%, respectively, which is consistent with the study findings of Corey et al. (32) and doPico et al. (33).

The present study found a significant association between coarse and fine dust particles and this result can be used in translating available measurements of dust size fractions. The coarse dust particles were the dominant size fraction measured in all studied areas and has been documented in previous studies (32–37).

Exposures to high short-term peak concentrations of flour dust may trigger respiratory symptoms. The peak exposure levels usually have a short-term character with duration usually ranged from 30 s to 4 min (9). Dough mixing as well as tipping and manual handling of flour are considered as the dustiest tasks (9), consistent with our highest peak concentration measured in the dough room. These peaks may contribute not only to time-weighted average exposure but can also play an important role in the advancement of awareness about potential adverse health effects (31). Since these exposure peaks correspond to relatively well-defined operations, the use of respiratory protection device during these activities should be recommended as demonstrated in the study by Heederik et al. (38).

This case report presents the first quantitative assessment of flour dust exposure in an Australian bakery factory. Further research is recommended on the respiratory hazards of bakery workers that would incorporate both dust exposure monitoring and respiratory health assessment including spirometry. It has been previously reported that flour is an organic fine particle with an array of allergens proteins (34, 35, 39). Additional information on specific allergens would provide further insights as previous studies have indicated that measurements of flour dusts are not sufficient as surrogate exposure measure of allergens found in flour (40, 41). Higher microbiological counts in the milling process may indicate equipment contamination and can contribute to bakery workers' microorganism exposures (34). Furthermore, lack of information on flour consumption in the bakery which may vary during certain work processes prevents a better interpretation of the study results, and this is acknowledged as a limitation.

The findings of the current case study underline the need of control measures to reduce dust exposure and avoid adverse health effects among workers. The best method of preventing adverse health outcomes would be to provide a working environment free from the hazards. While it may not be possible to eliminate dust exposures, its reduction to lower levels should be established as an ultimate target. One way is to modify the behavior of workers and the working practices. Previous studies have demonstrated that simple procedures such as emptying the bags of flour without shaking them, pouring the flour into the water and not vice versa, and cleaning the workplace using a vacuum cleaner instead of bristle broom seem to be effective to reduce the flour dust exposure (42, 43). Finally, education and training of workers are also important to curtail their exposure levels (4, 44). Regular cleaning and changing of working and protective clothes should be considered; facilities to wash hands (or take a shower) when leaving the workplace should be provided; eating, drinking, or smoking at the workplace should be avoided (1, 44, 45). Personal measures may focus on respiratory protection and personal protective equipment including clothes, gloves, and goggles; however, such control measures should be used as the last possible prevention measure.

## Conclusions

In this study bakery workers were exposed to high concentrations of airborne dust while at work, especially those working in the dough room whose measurements exceeded the recommended OEL and thus are susceptible to respiratory diseases. In addition to developing mechanisms for reducing their dust exposure, training, and education should be an effective strategy to adopt in the industry. The results obtained from the current study refer to a single bakery in Australia, and therefore do not allow for generalization to the entire population of Australian bakery workers.

## Data Availability Statement

The raw data supporting the conclusions of this article will be made available by the authors, without undue reservation.

## Ethics Statement

The studies involving human participants were reviewed and approved by Curtin University Human Research Ethics Committee (Approval Number HRE2017-0747). The patients/participants provided their written informed consent to participate in this study.

## Author Contributions

KR and YZ contributed to the conception and presentation of the case report. YZ conducted the data analysis. AL provided expertise and interpretation of data analysis. All authors contributed to manuscript writing, read, and agreed to the published version of the manuscript.

## Conflict of Interest

The authors declare that the research was conducted in the absence of any commercial or financial relationships that could be construed as a potential conflict of interest.

## Publisher's Note

All claims expressed in this article are solely those of the authors and do not necessarily represent those of their affiliated organizations, or those of the publisher, the editors and the reviewers. Any product that may be evaluated in this article, or claim that may be made by its manufacturer, is not guaranteed or endorsed by the publisher.
